# Diagnosis and treatment approaches for simultaneous onset of subarachnoid hemorrhage and thyroid storm: a case report

**DOI:** 10.1186/s12245-023-00490-4

**Published:** 2023-03-01

**Authors:** Aimi Ohya, Makoto Ohtake, Yusuke Kawamura, Taisuke Akimoto, Masayuki Iwashita, Tetsuya Yamamoto, Ichiro Takeuchi

**Affiliations:** 1 Department of Emergency and Critical Care, Yokohama City University Medical Center, Yokohama, Japan; 2 Department of Neurosurgery, Yokohama City University Medical Center, Yokohama, Japan; 3 Department of Neurosurgery, Yokohama City University Graduate School of Medicine, Yokohama, Japan

**Keywords:** Subarachnoid hemorrhage, Thyroid storm, Tachycardia

## Abstract

**Background:**

Subarachnoid hemorrhage and thyroid storm are similar in their clinical symptomatology, and diagnosis of these conditions, when they occur simultaneously, is difficult. Here, we report a rare case of concurrent subarachnoid hemorrhage and thyroid storm we encountered at our hospital.

**Case presentation:**

The patient was a 52-year-old woman. While bathing at home, the patient experienced a sudden disturbance of consciousness and was brought to our hospital. The main physical findings upon admittance were Glasgow Coma Scale score of E1V2M4, elevated blood pressure (208/145 mmHg), and tachycardia with atrial fibrillation (180 bpm) along with body temperature of 36.1 °C. Brain computed tomography revealed subarachnoid hemorrhage associated with a ruptured aneurysm of the posterior communicating artery branching from the left internal carotid artery, and aneurysm clipping was performed. Blood tests upon admission revealed high levels of free T3 and free T4 and low levels of thyroid-stimulating hormone. Upon determining that the patient had hyperthyroidism, thiamazole was administered. However, due to continuous impaired consciousness, fever, and persistence of tachycardia, the patient was diagnosed with thyroid storm. Oral potassium iodide and hydrocortisone were added to the treatment. The treatment was successful as the patient’s symptoms improved, and she became lucid.

In this case, we believe that in the presence of untreated hyperthyroidism, the onset of subarachnoid hemorrhage induced thyroid storm. Tachycardia of 130 bpm or higher, which is the diagnostic criterion for thyroid storm, rarely occurs with subarachnoid hemorrhage. Therefore, we believe it is an important factor for recognizing the presence of the thyroid storm. In this case, clipping surgery was prioritized which resulted in a favorable outcome. However, it is possible that invasive surgery may have exacerbated thyroid storm, suggesting that treatment should be tailored as per patient’s condition.

**Conclusion:**

If a pulse rate of 130 bpm or higher is observed alongside subarachnoid hemorrhage, we recommend considering the possibility of concomitant thyroid storm and testing for thyroid hormone. If concomitant thyroid storm is present, we believe that a treatment plan tailored to the patient’s condition is critical, and early diagnosis will lead to a favorable outcome for the patient.

## Background

Approximately 80–90% of subarachnoid hemorrhages are caused by ruptured cerebral aneurysms, and the case fatality rate at onset has been reported at 10–67% [1, 2]. Main symptoms are sudden headache, impaired consciousness, and vomiting. In addition, about 7–10% of patients with subarachnoid hemorrhage may experience heart failure symptoms with left ventricular wall dyskinesia [3]. In contrast, a thyroid storm refers to a pathological condition in which significant additional stress in the form of surgery, infection, and/or trauma occurs in the presence of an underlying, untreated, or poorly controlled thyroid-gland illness that initiates a breakdown of the compensatory mechanisms of the body against the impacts of thyroid hormone, resulting in a life-threatening condition [4, 5]. Irregular administration of antithyroid drugs, self-interruption, infection and trauma, surgery, ischemic heart disease, cerebrovascular accidents, and administration of iodine contrast media are the triggers. Previous reports on thyroid storm show that the case fatality rate is 50–90% when untreated and 10% or higher with appropriate treatment [6]. The following symptoms associated with thyroid storm are reported frequently: central nervous symptoms such as restlessness and impaired consciousness with a Glasgow Coma Scale (GCS) score of 14 or less (84.4% of patients); tachycardia of 130 beats per minute (bpm) or higher (76.2% of patients); gastrointestinal symptoms such as vomiting, diarrhea, and jaundice (69.5% of patients); fever of 38 °C (41.5% of patients); and symptoms of heart failure such as a New York Heart Association classification of 4 or a Killip classification of III or higher (39.5% of patients) [6, 7].

Regarding the treatment of subarachnoid hemorrhage, rebleeding and delayed cerebral vasospasms are important factors that worsen the prognosis of patients after the onset of subarachnoid hemorrhage. Conservative treatment of ruptured cerebral aneurysms results in a 20–30% occurrence rate of rebleeding in the first month, which exacerbates outcomes; therefore, early surgical intervention for the prevention of rebleeding within 72 h of onset is crucial [1, 8]. Reports suggest that surgery during the cerebral vasospasm stage has many complications, and thus, it is recommended that the preventive treatment for rebleeding after 72 h of onset should be performed after the cerebral vasospasm stage [9]. Surgical interventions to prevent rebleeding include craniotomy and endovascular treatment. The recommendations are to choose a treatment policy after a comprehensive assessment of the severity, site, and shape of the cerebral aneurysm along with treatment challenges, patient age, and other complications [2, 10]. In contrast, for the treatment of thyroid storm, in addition to the swift administration of antithyroid drugs, corticosteroids (hydrocortisone 300 mg/day or dexamethasone 8 mg/day) and potassium iodide should be administered simultaneously [6, 11]. *β*-blockers with β1 selectivity are the first-line treatment for tachycardia. In cases where the heart rate is 150 bpm or higher, the condition may become severe; therefore, treatment must be started proactively.

As discussed above, the clinical symptoms of subarachnoid hemorrhage and thyroid storm have much in common, and it is difficult to recognize thyroid storm, especially when subarachnoid hemorrhage and thyroid storm occur simultaneously. In this case report, we describe a case of subarachnoid hemorrhage and thyroid storm upon the first visit and discuss our diagnosis and treatment policy along with a review of the literature.

This study protocol was reviewed and approved by the Institutional Review Board, the Ethics Committee of the Yokohama City University Medical Center (approval no. B210400045). The study procedures were performed in accordance with the ethical standards laid down in the 1964 Declaration of Helsinki and its later amendments. Written informed consent was obtained from this patient for the publication of this case report.

## Case presentation

**Fig. 1:**
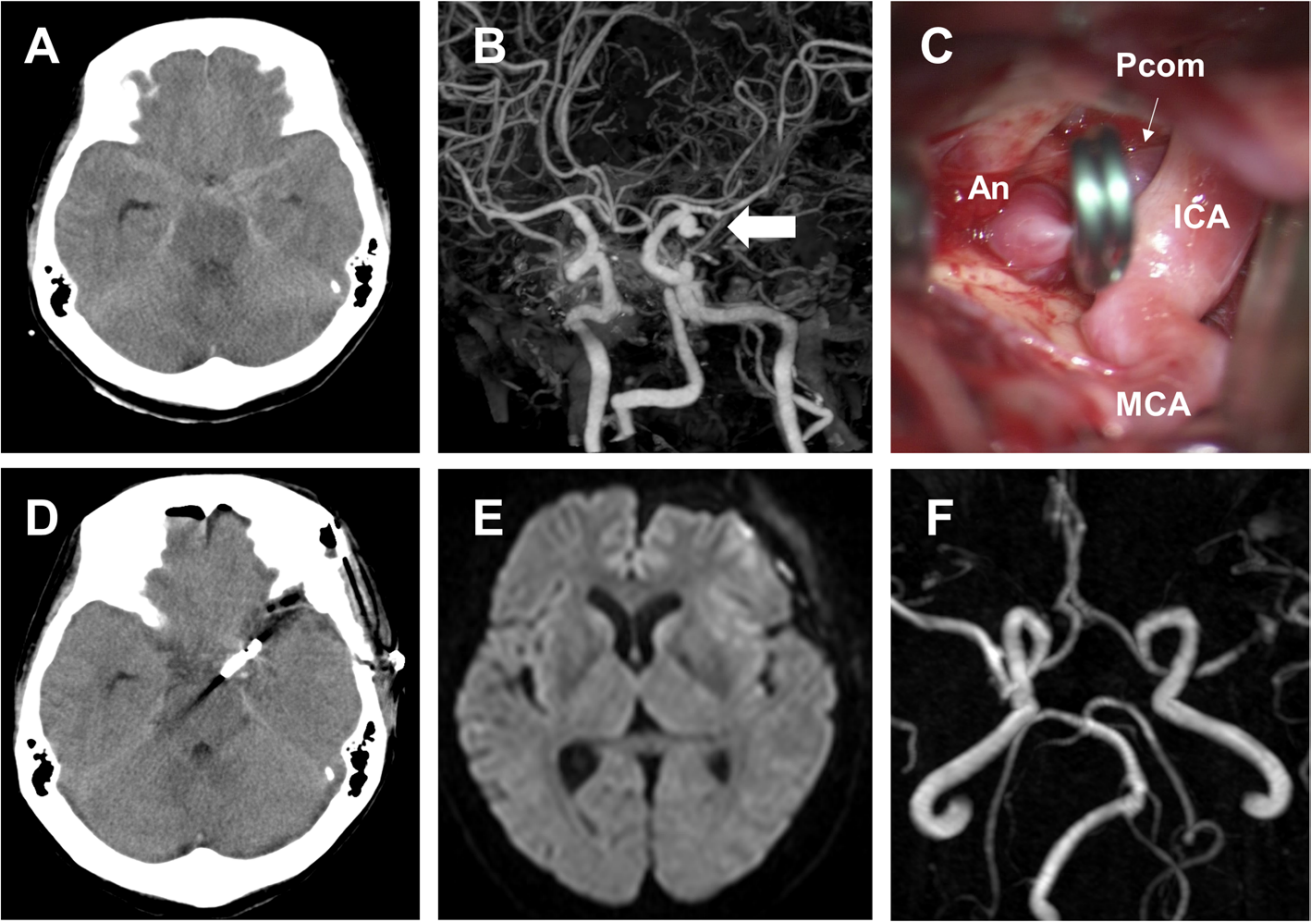
Imaging findings. **a** Computed tomography (CT) scan of the brain revealing subarachnoid hemorrhage. **b** CT angiographic view showing an aneurysm of the posterior communicating artery branching from the left internal carotid artery (*arrow*). **c** Intraoperative view showing a ruptured aneurysm* with bleb. **d** Postoperative axial CT imaging showing Sugita clip and no additional bleeding. **e** and **f** A diffusion-weighed magnetic resonance (MR) imaging and MR angiographical scan showing the absence of cerebral infarction or cerebral vasospasm. *An, aneurysm; ICA, internal carotid artery; MCA, middle cerebral artery; Pcom, posterior communicating artery

**Fig. 2:**
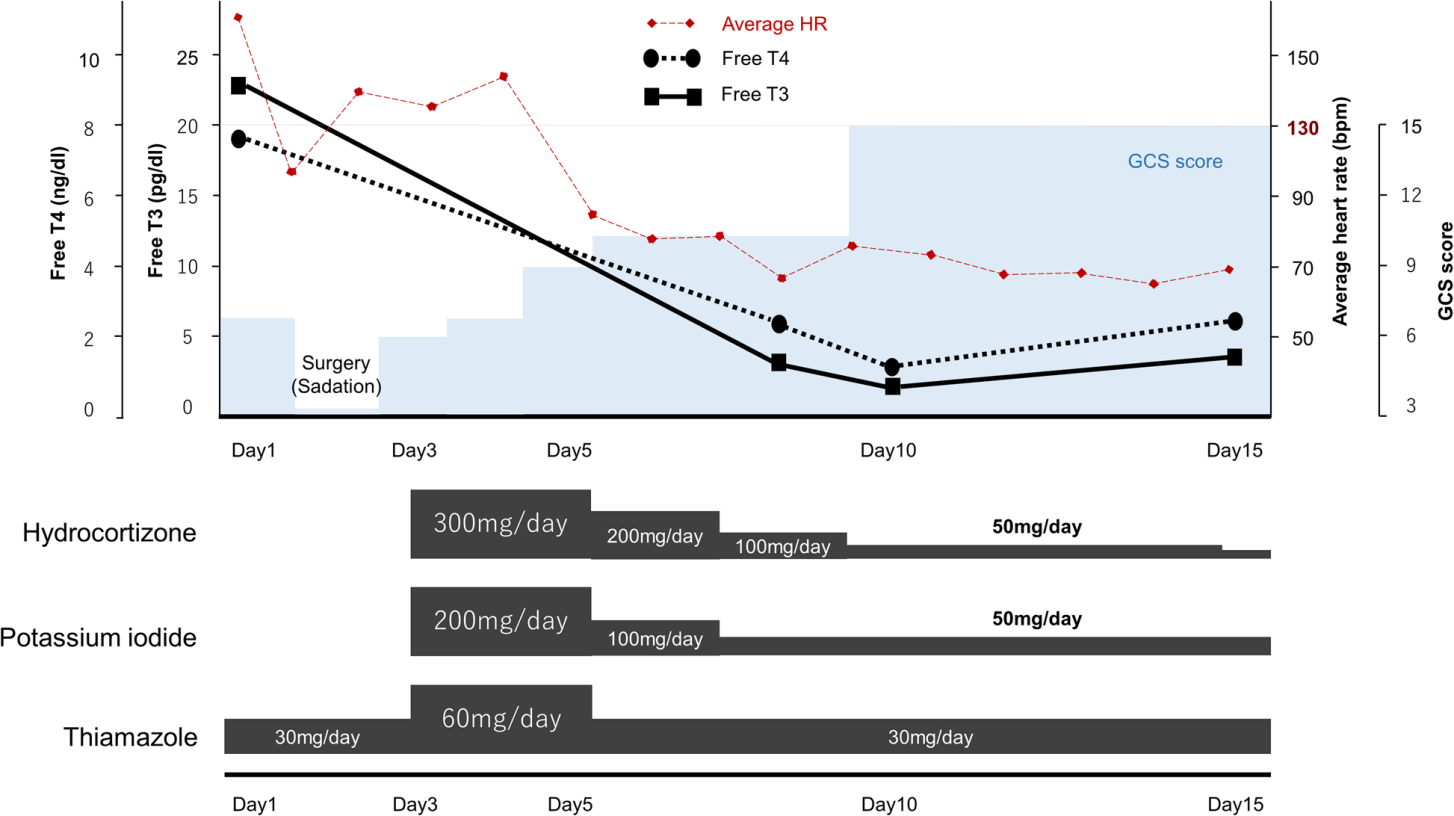
Progress chart documenting each clinical finding and drug dose. HR, heart rate; GCS, Glasgow Coma Scale

A 52-year-old woman with no medical history experienced impaired consciousness while bathing and was brought to our hospital. The primary physical findings upon her admission were a GCS score of E1V2M4, elevated blood pressure (208/145 mmHg) and tachycardia with atrial fibrillation (180 bpm), respiratory rate of 30 breaths/min, and body temperature of 36.1 °C. Brain computed tomography (CT) revealed subarachnoid hemorrhage (Fisher group 2, World Federation Neurological Surgeons; WFNS Grade IV) associated with a ruptured aneurysm of the posterior communicating artery branching from the left internal carotid artery (Fig. 1 a, b). A thyroid hormone test was performed since tachycardia was also observed. High values of free T3 (23.10 pg/dl) and free T4 (> 7.7 ng/dl) and low values of thyroid-stimulating hormone (*TSH* < 0.005 µIU/ml) were observed. As no thyroid dysfunction has been addressed previously, it was further determined to be a case of undiagnosed hyperthyroidism. In addition to the clinical symptoms of tachycardia and diffuse thyroid swelling on imaging, probable Graves’ disease was diagnosed based on high T3 and T4 levels, low TSH levels, and high thyrotropin receptor antibody levels (18.4 IU/l). Although tachycardia continued, vital signs were stabilized, and the aneurysm was irregular with a size of 8 mm and was accompanied by bleb. Therefore, clipping surgery was performed on the same day to prevent re-rupture (Fig. 1 c, d). The patient’s intraoperative vital signs remained stable. After the operation, oral thiamazole 30 mg/day and beta-blockers were initiated as the treatment for hyperthyroidism and tachycardia, respectively. However, disturbances of consciousness, fever (38.0 °C or higher), and tachycardia of 130 bpm or higher persisted. Thus, we diagnosed the patient as having a thyroid storm according to Japanese diagnostic criteria on the 3rd day [6, 7]. The dose of thiamazole was increased to 60 mg/day, and oral potassium iodide (200 mg/day) as well as hydrocortisone (300 mg/day) was added (Fig. 2). This treatment was successful, and on the 7th day, the patient’s free T3 decreased to 2.85 pg/dl, her heart rate improved to 100 bpm or less, and she became lucid. Brain magnetic resonance imaging (MRI) performed on the 14th day of hospitalization did not show findings suggestive of cerebral vasospasm or cerebral infarction (Fig. 1 e, f). Drug dosages were gradually tapered off as hydrocortisone was reduced to 30 mg/day on the 15th day, and thiamazole and potassium iodide were reduced to 30 mg/day and 50 mg/day, respectively, for continuous administration. The patient was transferred to a rehabilitation facility on the 20th day. Three months after the incident, the patient’s Glasgow Outcome Scale score indicated good recovery, and she achieved a modified Rankin Scale score of 1.

## Discussion and conclusions

### Characteristics of our case

We encountered a case of subarachnoid hemorrhage and thyroid storm upon admission. There are previous case reports of patients with hyperthyroidism who developed subarachnoid hemorrhage [12, 13]. However, to the best of our knowledge, none of these cases progressed to thyroid storm, and none has involved concomitant subarachnoid hemorrhage and thyroid storm, at the time of initial diagnosis. In this case, after the surgery, impaired consciousness and tachycardiac atrial fibrillation of 130 bpm or higher persisted, and we determined that the patient’s disturbances of consciousness were not solely due to subarachnoid hemorrhage. Eventually, the patient was diagnosed with thyroid storm [7]. Furthermore, it is possible that the patient was already in a state of thyroid storm since admission, and this state may have been exacerbated by the use of iodine contrast medium and surgical invasion [14]. However, since the results of the patient’s thyroid hormone tests were unavailable when a contrast-enhanced CT was performed on the first visit, we believe that contrast-enhanced evaluation was unavoidable. If we detect thyroid storm in advance, previous reports suggest that evaluation to confirm the cerebral aneurysms should be performed by head magnetic resonance angiography (MRA), without contrast-enhanced medium [15, 16]. However, MRA testing for patients with subarachnoid hemorrhage with unstable vital signs poses risks. We believe that evaluation according to the patient’s condition is the best option. In this case, we think that untreated hyperthyroidism, stress due to the onset of subarachnoid hemorrhage, and factors such as iodine contrast agent use resulted in a thyroid storm.

### Diagnosis for simultaneous subarachnoid hemorrhage and thyroid storm

Subarachnoid hemorrhage and thyroid storm share many common symptoms, and when they occur in a patient concomitantly, they are not easy to diagnose and have extremely high mortality rates [2, 6]. To prevent complications in these cases, it is important to make a reliable diagnosis upon admission or first visit and prioritize treatment. In this case, diagnosis of thyroid storm was delayed to the 3rd day of hospitalization, after aneurysm clipping was performed. Subarachnoid hemorrhage is relatively easy to diagnose due to imaging results in addition to episodes including headache and impaired consciousness. In contrast, diagnosis of thyroid storm is often difficult because it has many symptoms like subarachnoid hemorrhage. It often develops in patients with undiagnosed thyroid disease at the time of first visit. Therefore, if subarachnoid hemorrhage is diagnosed first, we believe it can be extremely difficult to diagnose concurrent thyroid storm. Although thyroid function tests are important for diagnosis, it is impractical to perform thyroid function tests in all subarachnoid hemorrhage cases. Thus, it is necessary to screen patients who should undergo thyroid function testing when they present with subarachnoid hemorrhage. Thyroid function tests should be performed for patients with typical symptoms of hyperthyroidism such as exophthalmos and abnormal sweating or for patients with a history of thyroid disease. Furthermore, when the typical symptoms of hyperthyroidism are subtle or absent, it is difficult to make a definitive assessment. We thus focus on the existence of tachycardia.

In subarachnoid hemorrhage, more than 90% of patients exhibit abnormal electrocardiogram findings such as sinus bradycardia, sinus tachycardia, ST elevation, and QT prolongation [17, 18]. Summarizing past data on heart rate, the average heart rate was 75–86 bpm, and approximately 20% of patients exhibit tachycardia of 100 bpm or more [13, 19, 20]. However, tachycardia of 130 bpm or more is rarely reported. Among 128 cases with aneurysmal subarachnoid hemorrhage between January 2013 and November 2021 at our hospital, the average heart rate at admittance was 83.8 ± 19.5 bpm (range, 40–180), and 23 patients (17.9%) had a heart rate of 100 or more. It is important to note that none of these patients, except the patient presented in this report, who was the only patient diagnosed with thyroid storm, had tachycardia above 130 bpm. However, in thyroid storm cases, approximately 80% of the patients have exhibited tachycardia exceeding 130 bpm, which is the diagnostic criterion for thyroid storm [6, 7]. Thus, when tachycardia of 130 bpm or more is observed in patients with subarachnoid hemorrhage, we recommend performing a thyroid function test to differentiate thyroid storm.

### Treatment policy for simultaneous subarachnoid hemorrhage and thyroid storm

Clear evidence on which disease should be prioritized with respect to treatment is absent when a combination of subarachnoid hemorrhage and thyroid storm are diagnosed together. When surgery for subarachnoid hemorrhage is prioritized, surgical invasion may exacerbate the thyroid storm and worsen a patient’s condition. Delayed surgery for subarachnoid hemorrhage may pose a risk of rebleeding and may lead to a fatal outcome. In previous reports, when thyroid storm treatment was prioritized over gastrointestinal and respiratory diseases, the resulting outcomes were good [11, 21]. In contrast, when subarachnoid hemorrhage is conservatively treated, the rebleeding rate is 3–4% on the first day of onset, 1–2% per day during the following 4 weeks, and 20–30% in the first month [1, 22]. Poor outcomes during rebleeding episodes have been reported too. In this case, treatment for thyroid storm began a few days after aneurysm clipping surgery due to the delayed diagnosis, and symptoms were improved within a few days with a desirable outcome. We believe that prioritization of surgical intervention for subarachnoid hemorrhage is dependent on comprehensive assessment of the urgency of surgery based on the size and shape of the cerebral aneurysm and the presence of hematoma formation. Craniotomy and endovascular treatment are options to prevent rebleeding, but the iodine contrast medium used in endovascular treatment has a high potential of exacerbating thyroid storm. Thus, craniotomy seems to be the first choice. When thyroid storm treatment is prioritized, it is recommended to pay close attention to rebleeding and prevention of cerebral vasospasm by sedation and antihypertensive therapy and to perform surgical intervention promptly when thyroid function is stable, and the cerebral vasospasm period is over.

This study had some limitations. Because this is a study of a single case of concomitant subarachnoid hemorrhage and thyroid storm, which has rarely been previously reported, sufficient literature is unavailable. Since the concomitant occurrence of these diseases is quite difficult to diagnose, it is likely that in several previous cases, thyroid storm was overlooked with a poor outcome among patients. Fortunately, we were able to achieve a good outcome in this case. However, this is ultimately a report of a single case, and treatment priorities and other aspects of treatment policies should be individually considered for each case. It is necessary to consider thyroid storm as a differential diagnosis when a patient presents with subarachnoid hemorrhage accompanied with tachycardia. A thyroid hormone test should be performed in such cases. In the future, we hope that this report will help diagnose thyroid storm in subarachnoid hemorrhage cases with tachycardia among as many patients as possible, and that more patients will have better outcomes.

In conclusion, in patients with subarachnoid hemorrhage who present with tachycardia of 130 bpm or higher, the possibility of concomitant hyperthyroidism should be considered first followed by thyroid hormone tests. We suggest that whenever subarachnoid hemorrhage and thyroid storm occur concomitantly, patient-tailored treatment plans should be considered, taking into account the urgency and acuteness of each patient’s condition. This will lead to improved outcomes among such patients.

## Data Availability

Data sharing is not applicable to this article as no datasets were generated or analyzed during the current study.

## Abbreviations

GCS: Glasgow coma scale
Bpm: Beats per minute
CT: Computed tomography
WFNS: World federation of neurosurgical societies
TSH: Thyroid-stimulating hormone
MRI: Magnetic resonance imaging
MRA: Magnetic resonance angiography
